# Suture-Button Repair Improves Outcomes in Arthroscopically Diagnosed Chronic Syndesmotic Injury Despite Low Imaging Sensitivity

**DOI:** 10.1016/j.asmr.2025.101227

**Published:** 2025-07-29

**Authors:** Tarjei Vinje, Eivind Inderhaug, Jon Jarning, Randi M. Hole, Nils Vetti, Merete A. Malt, Kjell Matre

**Affiliations:** a Department of Orthopedic Surgery, Haukeland University Hospital, Bergen, Norway; b Department of Clinical Medicine, University of Bergen, Bergen, Norway; c Norwegian Arthroplasty Register, Department of Orthopedic Surgery, Haukeland University Hospital, Bergen, Norway; d Department of Radiology, Haukeland University Hospital, Bergen, Norway; e Department of Physiotherapy, Haukeland University Hospital, Bergen, Norway

## Abstract

**Purpose:**

To evaluate patient-reported outcomes after arthroscopic suture-button repair of chronic isolated syndesmotic injuries and to compare the diagnostic sensitivity of standard imaging with arthroscopy.

**Methods:**

Patients with chronic (≥3 months) syndesmotic injuries confirmed by arthroscopy (≥2 mm widening) who underwent suture-button repair between 2017 and 2021 were included. Exclusion criteria were ankle fractures, previous major surgery, or significant arthritis. Foot Function Index and Foot-and-Ankle Ability Measure scores were recorded preoperatively as part of routine clinical assessment and at final follow-up. The minimal clinically important difference was defined as 75% of preoperative standard deviation.

**Results:**

Forty-one patients (43 ankles) were analyzed. Median age was 32 years (15-57 years), 63% were female, and time from injury to surgery was 1.7 years (0.25-25). Fifteen ankles (35%) received concomitant lateral suture-tape stabilization. Arthroscopic syndesmotic widening was 2-3 mm in 5%, 3-4 mm in 10%, >4 mm in 46%, and posterior in 38%. All 6 Foot Function Index and Foot-and-Ankle Ability Measure subscores improved significantly (*P* < .001), with 79% to 93% achieving minimal clinically important difference at a median 2.3-year follow-up (1.0-4.8). Sensitivity for syndesmotic pathology was 5% for radiographs and stress fluoroscopy, 22% for computed tomography, and 26% for magnetic resonance imaging.

**Conclusions:**

Arthroscopic suture-button repair led to significant improvements in patient-reported outcomes for chronic isolated syndesmotic injuries. Standard imaging had low sensitivity, and a posterior-predominant syndesmotic widening pattern was commonly observed arthroscopically. These findings suggest that chronic syndesmotic instability is underdiagnosed, highlight the role of arthroscopic evaluation for accurate diagnosis, and support the use of suture-button stabilization as an effective treatment.

**Level of Evidence:**

Level IV, therapeutic case series.

Syndesmotic injuries represent a hidden challenge in ankle injury management. They account for up to 25% of ankle sprains, with an incidence of 50 to 500 per 100,000 annually.^1^, ^2^, ^3^, ^4^ Compared with lateral ankle sprains, syndesmotic injuries typically produce more severe and prolonged symptoms.^5^^,^^6^ Although many resolve within days to weeks, some persist for 3 to 6 months, even with early detection and targeted rehabilitation.^7^ Patients whose symptoms persist beyond this period and never return to preinjury function remain poorly studied, largely because occult syndesmotic instability is difficult to identify.^8^

Diagnostic difficulty arises from symptom overlap with lateral sprains and the limited accuracy of standard imaging modalities.^9^, ^10^, ^11^, ^12^ As clinical signs become increasingly nonspecific over time, persistent dysfunction is often misattributed to unrelated pathology.^13^ Radiographs and computed tomography (CT) frequently miss subtle or dynamic diastasis, and magnetic resonance imaging (MRI) may appear normal once chronic scarring bridges the ligaments.^14^^,^^15^ Measuring radiologic miss-rates in chronic injuries can refine diagnostic algorithms and guide when to proceed with arthroscopic examination, the gold standard that is too invasive for routine screening.^16^^,^^17^

At our center, we reserve diagnostic arthroscopy for patients with more than 3 months of disabling symptoms and a history strongly suggestive of syndesmotic injury. This 3-month threshold balances the time needed for spontaneous recovery against the risk of prolonged disability if recovery fails. In those with arthroscopically confirmed instability, we perform suture-button fixation, a technique well established for acute fracture–related syndesmotic injuries, yet less defined in chronic isolated injuries.^13^^,^^18^

The purposes of this study were to evaluate patient-reported outcomes after arthroscopic suture-button repair of chronic isolated syndesmotic injuries and to compare the diagnostic sensitivity of standard imaging with arthroscopy. We hypothesized that suture-button repair would improve patient-reported outcomes and standard imaging would have poor sensitivity for detecting these injuries.

## Methods

### Study Design and Participants

**Fig 1:**
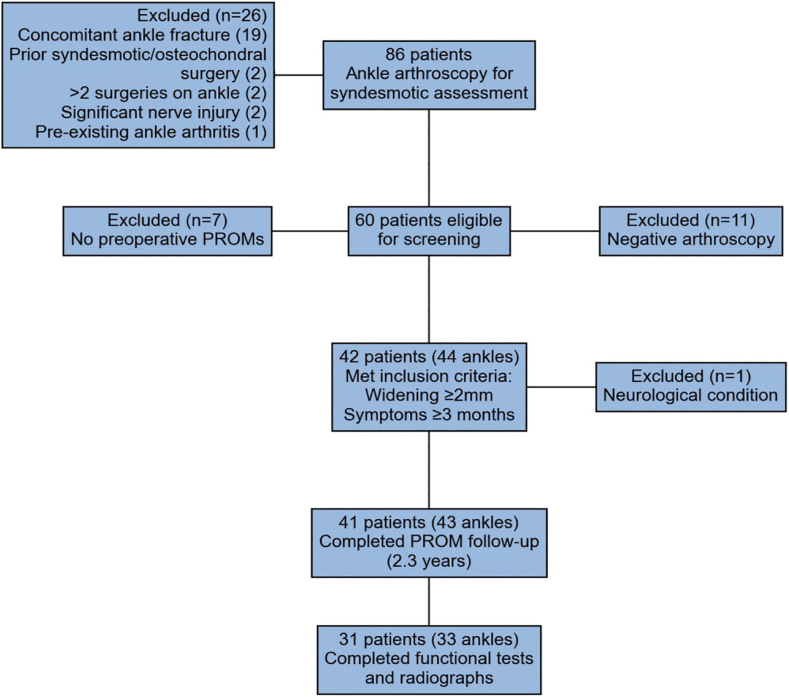
Flow diagram of patient selection. Initial arthroscopic assessment for syndesmotic instability was performed in 86 patients. After applying exclusion criteria and confirming syndesmotic widening ≥2 mm with persistent symptoms for ≥3 months, 41 patients (43 ankles) completed postoperative patient-reported outcome measures (PROMs) at median follow-up of 2.3 years. A subset of 31 patients (33 ankles) completed additional functional testing and radiographic assessment.

This retrospective cohort study was conducted at a single academic center between March 2017 and January 2021. All patients who underwent anterior ankle arthroscopy to assess possible syndesmotic instability were screened (Fig 1). Inclusion criteria were arthroscopically confirmed syndesmotic widening ≥2 mm, persistent symptoms for ≥3 months postinjury, documented preoperative patient-reported outcome measures (PROMs), and at least 1 year of postoperative follow-up.

Exclusion criteria included concomitant ankle fracture, previous syndesmotic or osteochondral surgery, more than 2 previous operations on the affected ankle, significant preoperative nerve injury, and pre-existing ankle arthritis. The Regional Ethics Committee approved the study (approval no. [264009]), and all participants signed an informed consent.

### Clinical and Radiologic Evaluation

Patients were generally referred because of severe, persistent ankle pain and markedly reduced function after trauma. Often, patients could not recall a clear injury event until questioned in detail. When described, injury mechanisms included not only the classical external-rotation pattern but also supination or axial-load trauma, and sometimes remained unclear. On prompting, many patients recalled acute signs such as a distinct "pop," significant early swelling, bruising (laterally, medially, or proximally), and prolonged inability to bear weight. Many had initial radiography to exclude a fracture. Chronic symptoms typically involved persistent swelling, push-off weakness, stiffness, instability (giving-way), and pain that could be lateral, medial, anterior, or diffuse.

For this study, we extracted clinical signs explicitly documented in patient records: tenderness over the anterior or posterior tibiofibular ligaments, and a positive Cotton test, defined as palpable side-to-side laxity or ≥2 mm lateral talar translation observed during mortise fluoroscopy.^19^

Preoperative imaging including unilateral non−weight-bearing radiographs, bilateral weight-bearing radiographs, CT scans, or MRI was performed at the surgeon’s discretion. Diagnostic conclusions were determined on the basis of finalized musculoskeletal radiology reports from various radiologists and institutions; original images were not re-evaluated.

Contemporary standards for syndesmotic assessment cite specific thresholds for abnormality. For instance, radiographic evaluations, with measurements taken 10 mm above the tibial plafond may identify a tibiofibular clear space >6 mm (on the anteroposterior view), tibiofibular overlap <6 mm (anteroposterior view) or <1 mm (mortise view), or medial clear space >4 mm as abnormal findings. On bilateral weight-bearing radiographs, a side-to-side difference >1 mm in these parameters was also considered indicative of instability. Typical CT criteria define a side-to-side difference >2 mm in the tibiofibular interval as abnormal, with measurements taken from axial slices 10 mm above the tibial plafond. MRI may identify ligament discontinuity, abnormal morphology (such as waviness or thickening), or increased signal intensity within the syndesmotic ligaments.^9^^,^^14^^,^^15^

However, as reports originated from multiple radiologists and institutions, the precise diagnostic criteria applied in each case were not investigated. This was underscored by observed inconsistencies; for example, some CT reports identified side-to-side differences of 1 to 2 mm as pathological, indicating some heterogeneous practice. Because isolated clinical tests and routine imaging may be inconclusive, or even normal, once an injury becomes chronic, arthroscopy was offered when three criteria were met: (1) a history suggestive of syndesmotic trauma, (2) persisting major symptoms (pain with markedly limited function) for more than three months, and (3) no alternative diagnosis that better explained the symptoms. Immediately before arthroscopy, fluoroscopic stress views estimated talar tilt (0–5°, >5-15°, or >15°) and any syndesmotic or medial clear space widening (>2 mm).

### Surgical Procedure and Postoperative Care

All operations were performed with the patient supine under total intravenous anesthesia without routine muscle relaxation. A thigh tourniquet, applied preoperatively before draping, was typically inflated only after the diagnostic arthroscopy portion and immediately before suture-button placement.

Anterior ankle arthroscopy was performed without joint distraction, using a standard 4.0-mm 30° arthroscope introduced through an anteromedial viewing portal.^20^ Instruments were passed via an anterolateral working portal. Fluid pump pressure was maintained at 25 to 35 mm Hg. The tibiofibular interval was probed dynamically with a 4-mm trocar, a 4-mm shaver, or a 2- to 4-mm hook probe to establish the minimal central gap, in a manner consistent with principles described by Guyton et al.^17^ The arthroscope itself was occasionally leveraged to tilt the talus carefully to aid visualization of widening, avoiding iatrogenic cartilage damage. No manual external-rotation stress was applied to the foot during this assessment. Syndesmotic widening of ≥2 mm was considered pathologic. Ankles in which posterior widening was observed to clearly exceed the central widening were classified as having a posterior-predominant pattern.

When widening met the ≥2 mm threshold, scar tissue within the syndesmotic interval was excised. To restart the healing process in ligaments that had scarred in an elongated state, the anteroinferior tibiofibular ligament was typically divided centrally with scissors. The posteroinferior tibiofibular ligament was partially released superiorly from its tibial attachment with a 4.0 shaver (Bone Cutter 4.0; Arthrex, Naples, FL), preserving its distal-most fibers to guide healing in an anatomically reduced position. In some ankles with minimal talar tilt, accessing the full extent of the posterior syndesmosis for release could be challenging.

Stabilization was achieved with either 2 identical suture-buttons or a hybrid combination (TightRope, Arthrex; INVISIKNOT Non-Fracture, Smith & Nephew, Andover, MA).^21^ These were inserted through separate 3 to 5 cm longitudinal lateral and medial incisions. The first tunnel was drilled from the fibula, just proximal to the syndesmosis, aiming slightly more proximal on the tibia; the second tunnel was placed approximately 1 to 2 cm above the first. Care was taken during lateral drilling to preserve an anterior fibular bony bridge. The medial incision was guided by the marked exit of the drill tip and facilitated direct visualization of the tibial exit points, allowing for protection of the saphenous vein and nerve from impingement. Correct and firm button seating against the cortex were confirmed by fluoroscopy, by direct visualization and palpation. Three reinforcing knots were typically tied over the most proximal button after solid sequential tightening, even if the suture-button was "knotless." Arthroscopic reassessment of syndesmotic reduction after final tightening was not routinely performed, but compression of the syndesmosis was crudely assessed on fluoroscopy comparing side to side before and after suture button compression.

A modified Broström procedure with suture-tape augmentation was added if intraoperative stress fluoroscopy showed talar tilt visually estimated to be 10 to 15° or greater.^22^ This combined procedure also was performed if lateral ankle instability had been the primary preoperative diagnosis but syndesmotic widening (≥2 mm) was identified arthroscopically, even if the visually estimated talar tilt appeared to be less than 10 to 15°.^22^

We used no casting or bracing postoperatively. Patients bore limited weight (10-20 kg) for 3 weeks before progressing to full weight-bearing as tolerated. Follow-up visits were scheduled at 6 weeks and 3 months. Suture-button removal was considered if patients reported discomfort, generally no earlier than one year postoperatively.

### Outcome Measures and Follow-Up Assessment

The primary outcome was the change in Foot Function Index (FFI) and Foot and Ankle Ability Measure (FAAM) scores from preoperative levels to at least 1 year postoperatively. Preoperative PROMs were obtained by the first author during the diagnostic process, completed together with the patients, and documented in the electronic patient record. The FFI assesses pain and functional limitations using a 17-item scale scored from 0 to 10, whereas the FAAM evaluates limitations in activities of daily living (21 items) and sports activities (7 items) using Likert scales (0-4) converted to 0 to 100. Each measure also includes a global score, an FFI limitation score (0-10) and FAAM-ADL and FAAM-Sport function scores (0-100), yielding a total of 6 PROM subscores.

At least 1 year postoperatively, patients completed follow-up PROM assessments via telephone interviews conducted by an independent assessor (J.J.) not involved in the surgical care. During these interviews, patient satisfaction was also recorded on a 0 to 10 scale categorized as very dissatisfied (0-2), dissatisfied (3-4), neutral (5-6), satisfied (7-8), or very satisfied (9-10). All follow-up PROM and satisfaction data were documented in the electronic patient record.

Secondary outcomes included suture-button removal rates and complications, obtained from the electronic patient journal. A subset of patients returned for functional and radiographic assessment conducted by independent clinicians (J.J., M.A.M.) not involved in the surgery (Fig 1). These evaluations occurred shortly after the PROM follow-up assessments and included single-leg stance balance, heel raise endurance, and ankle dorsiflexion range of motion. Bilateral weight-bearing ankle radiographs were also obtained at this time to assess tibiotalar and medial clear space, and anterior tibial bony spur formation.

### Data Collection and Statistical Analysis

Pre- and postoperative PROMs, along with demographic, injury, surgical, and complication data were retrieved from the electronic patient journal (EPJ; DIPS, Bodø, Norway). Imaging records were accessed via the Picture Archiving and Communication System (Sectra, Linköping, Sweden), and fluoroscopic stress test results were documented in the EPJ. Body mass index, American Society of Anesthesiologists status, and surgical time were recorded in the surgical planning program Orbit (Evry, Oslo, Norway). Double data entry ensured accuracy of PROM and satisfaction scores.

Sensitivity of imaging modalities was calculated by dividing the number of positive findings for syndesmotic pathology on each modality by the total number of ankles examined with that modality, using arthroscopic confirmation of ≥2 mm widening as the reference standard.

Statistical analyses were performed using R version 4.2.3 (R Foundation for Statistical Computing, Vienna, Austria) and Python 3.12 (Python Software Foundation), with *P* < .05 considered significant. Before applying paired *t* tests, normality of data distributions was assessed using the Kolmogorov-Smirnov test. If distributions were non-normal, Mann-Whitney *U* tests were used. Missing data were minimal; no imputation was performed, and analyses were determined using available data only. No formal power analysis was conducted as this was a retrospective study. The minimal clinically important difference (MCID) was defined as 75% of preoperative score standard deviation, rather than the conventional 50%,^23^ due to the lack of established MCID values for the 6 PROMs in chronic syndesmotic instability.^23^ This more stringent threshold increases confidence in the clinical significance of observed improvements.

## Results

Of 86 patients initially screened, 41 patients (43 ankles) with chronic isolated syndesmotic injuries met inclusion criteria and where analyzed (Fig 1). The cohort was predominantly female (27/43, 63%), with a median age of 32 years (range 15-57 years). Median time from injury to surgery was 1.7 years (range 0.25-25 years). Preoperative clinical tests were documented in 34 ankles; 29 of 34 (85%) showed at least 1 positive sign, most commonly anterior-syndesmosis tenderness (21/34, 62%), Table 1.

**Table 1:** Clinical and Diagnostic Characteristics of 41 Patients (43 Ankles)

Category	Value
Age, yr, median [range]	31.6 [15.2-57.1]
Sex, female, n (%)	27 (62.8)
Side, right, n (%)	21 (48.8)
ASA classification, n (%)	
1	23 (53.5)
2	19 (44.2)
3	1 (2.3)
BMI, mean ± SD	28.2 ± 5.57
Normal weight (18.5-24.9), n (%)	13 (30.2)
Overweight (25-29.9), n (%)	19 (44.2)
Obese (≥30), n (%)	11 (25.6)
Time from injury to surgery, yr, median [range]	1.69 [0.25-25.3]
Surgical duration	
Suture button alone, min, mean ± SD	57.3 ± 14.6
With internal brace, min, mean ± SD	92.6 ± 15.2
Follow-up duration, yr, median [range]	2.26 [1.01-4.79]
Clinical syndesmotic tests (n = 34), n (%)	
Anterior syndesmosis tenderness	21 (61.8)
Posterior syndesmosis tenderness	15 (44.1)
Cotton test	13 (38.2)
Imaging modality, weight-bearing radiography positive, n/N (%)	1/21 (4.8)
CT 1-2 mm widening	3/23 (13.0)
CT 2-3 mm widening	2/23 (8.7)
MRI syndesmotic injury	10/38 (26.3)
Fluoroscopic assessment (n = 39), n (%)	
Talar tilt 0-5°	22 (56.4)
Talar tilt 5-15°	6 (15.4)
Talar tilt >15°	11 (28.2)
Syndesmotic widening, n (%)	2 (5.1)
Medial clear space widening, n (%)	10 (25.6)
Arthroscopic findings (n = 39), n (%)	
2-3 mm widening	2 (5.1)
3-4 mm widening	4 (10.2)
>4 mm widening	18 (46.2)
Predominantly posterior	15 (38.5)

NOTE. Values for continuous variables are presented as median [range] or mean ± SD. Categorical variables are presented as counts (percentages). For clinical tests and fluoroscopic assessments, numbers in parentheses (n) indicate the total ankles examined. For imaging modalities, n/N represents the number of positive findings out of the total ankles examined. Results from unilateral radiography (n = 13) and unilateral CT (n = 9) are not shown, as no syndesmotic widening was detected in these examinations. Radiographic interpretations were determined by initial radiologist reports, except for stress fluoroscopy. Arthroscopic findings were specified in 39 ankles, whereas 4 ankles had unspecified syndesmotic widening (≥2 mm).

ASA, American Society of Anesthesiologists; BMI, body mass index; CT, computed tomography; MRI, magnetic resonance imaging; SD, standard deviation.

**Fig 2:**
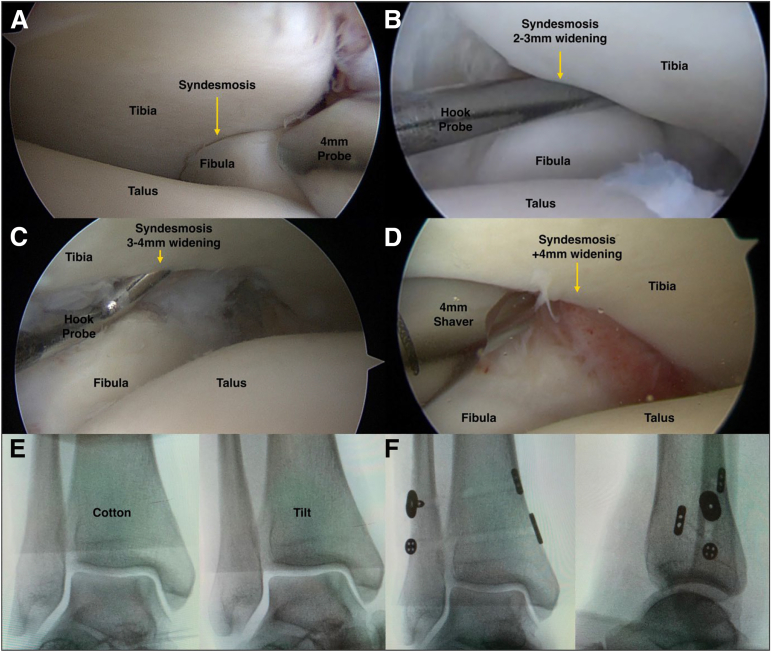
Arthroscopic views (patient is supine, anteromedial viewing portal, 30° arthroscope) showing grades of syndesmotic widening: (A) normal (left ankle), (B) 2- to 3-mm widening (right ankle), (C) 3- to 4-mm widening (right ankle), and (D) over 4-mm widening (right ankle). Images C, E, and F track the journey of a patient initially treated for a suspected anterolateral impingement through arthroscopic debridement of synovitis. During this initial procedure, syndesmotic widening was identified. However, this primary soft-tissue treatment did not improve the patient's symptoms, prompting a return for a stabilization procedure using suture-button. Although the Cotton test was negative for syndesmotic and medial widening, and the talar tilt was 0-5° at the second surgery, the patient experienced issues with instability, picture e. After careful debridement centrally in the syndesmosis, without touching the anterior or posterior syndesmosis, a widening graded 3-4 mm was found (C). For stabilization, a combination of implants was used—the TightRope Syndesmosis (Arthrex) and the INVISIKNOT Non-fracture (Smith & Nephew) (F). This led to an improvement in symptoms. However, the suture-tapes were eventually removed due to resulting stiffness and pain.

Arthroscopic examination detailed widening in 39 ankles: 2 to 3 mm in 2 of 39 (5%), 3 to 4 mm in 4 of 39 (10%), >4 mm in 18 of 39 (46%). A posterior-predominant “reverse open-book” pattern was noted in 15 of 39 (38%). Four additional ankles from the total cohort recorded widening ≥2 mm without further specification (Table 1 and Fig 2).

Lateral stabilization with a modified Broström procedure was added in 15 of 43 ankles (35%): 11 of 11 ankles with talar tilt >15°, 4 of 6 with 5 to 15°, and 0 of 22 with 0 to 5°. Mean operative time was 57 minutes (standard deviation, 15 minutes) for isolated syndesmotic repair and 93 minutes (standard deviation, 15 minutes) when a Broström was added.

**Fig 3:**
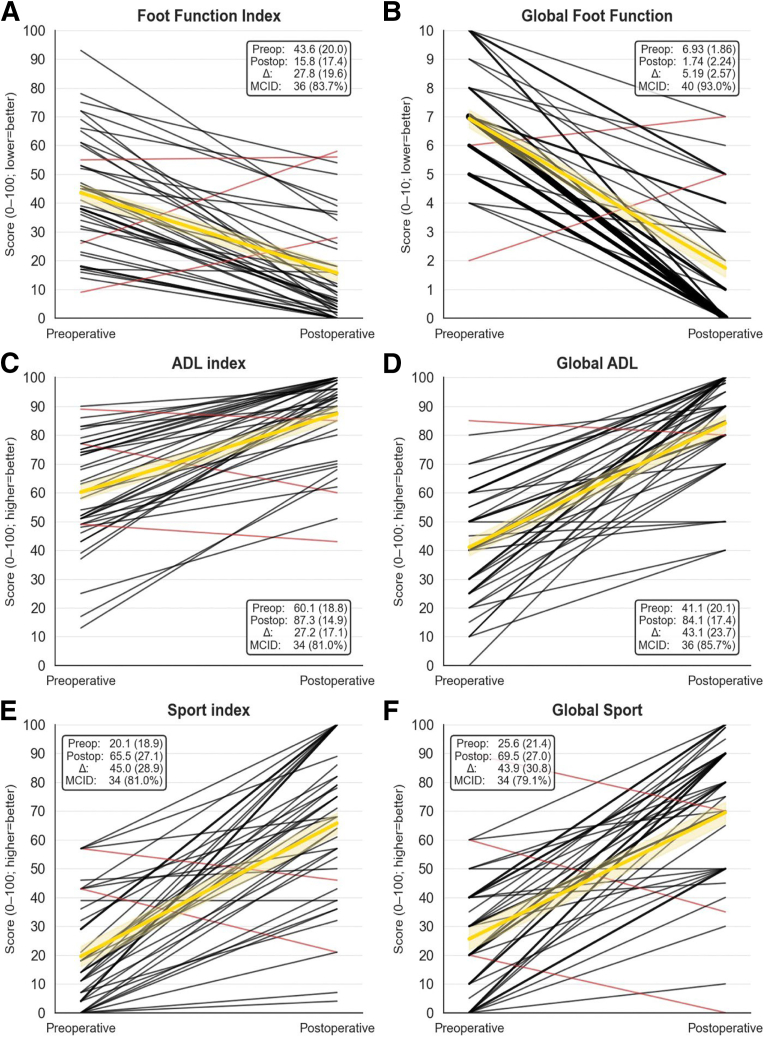
Spaghetti plots illustrating individual patient trajectories of Patient-reported outcome measures (PROMs) before and after suture-button repair for chronic syndesmotic injuries. The assessed PROMs include: Foot Function Index (FFI): (A) FFI index (17 items, 0–100 scale, lower = better); (B) Global FF (1 item, 0–10 scale, lower = better). Foot and Ankle Ability Measure (FAAM), all on a 0–100 scale (higher = better): (C) Activities of Daily Living (ADL) index (21 items); (D) Global ADL (1 item); (E) Sport index (7 items); (F) Global Sport (1 item). Each patient's pre- to postoperative course is represented by a line. Black lines indicate stable or improved scores, while red lines show deterioration. Thicker lines denote multiple patients sharing similar trajectories, increasing line thickness by adding 50% of the original line thickness for each additional patient. The gold line represents the mean trajectory, with a semi-transparent band illustrating the mean ±1 standard error. Sample size was 43 ankles for all measures, except n = 42 for the Activities of Daily Living (ADL) index/global and Sport index. Inset boxes show preoperative (Preop), postoperative (Postop), and change (Δ) scores as mean (standard deviation), and minimal clinically important difference (MCID) achievement as n (%). MCIDs, defined as 75% of the preoperative standard deviation, were: FFI index (15 points), FFI global (1.4 points), ADL index (14 points), ADL global (15 points), Sport index (14 points), and Sport global (16 points). All PROMs improved significantly (paired *t* tests, *P* < .001).

**Fig 4:**
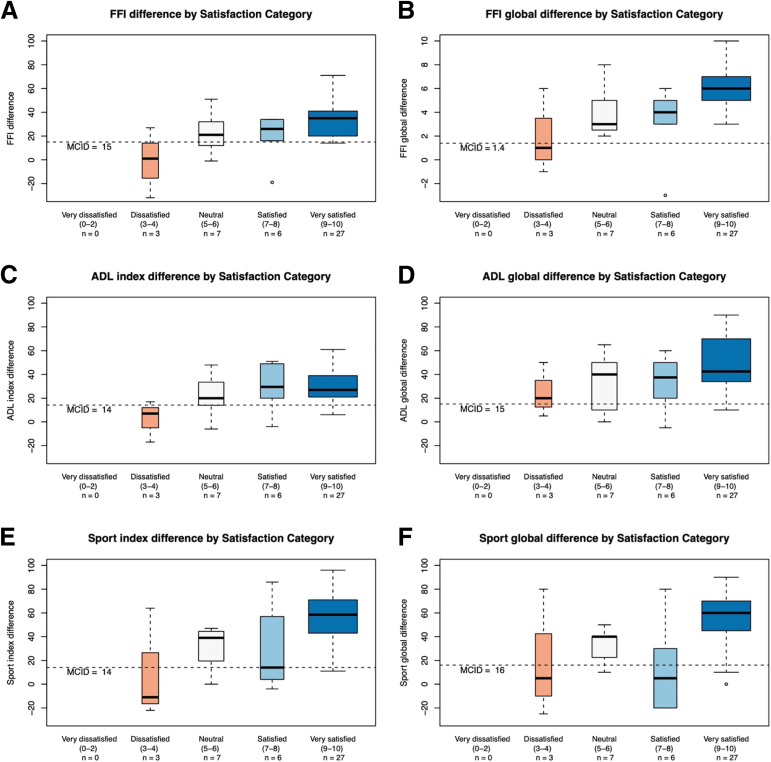
Box plots illustrating changes in patient-reported outcome measures (PROMs) after suture-button repair for chronic syndesmotic injuries, categorized by patient satisfaction levels. The assessed PROMs include: Foot Function Index (FFI): (A) FFI index (17 items, 0-100 scale, lower = better); (B) FFI global (1 item, 0-10 scale, lower = better). Foot and Ankle Ability Measure (FAAM), all on 0-100 scale (higher = better): (C) ADL index (21 items); (D) Activities of Daily Living (ADL)global (1 item); (E) Sport index (7 items); and (F) Sport global (1 item). A positive change score indicates improvement from preoperative to postoperative status. Patient satisfaction was recorded on a 0-10 scale and categorized as: “Very dissatisfied” (0-2, n = 0 ankles), “Dissatisfied”(3-4, n = 3 ankles), “Neutral” (5-6, n = 7 ankles), “Satisfied” (7-8, n = 6 ankles), and “Very satisfied” (9-10, n = 27 ankles). Sample size was 43 ankles for all measures, except n = 42 for the ADL index/global and Sport index. Box plots display the median (central line), interquartile range (box boundaries representing the 25th and 75th percentiles), and data range (whiskers extending to the most extreme data point within 1.5 times the interquartile range). Outliers are shown as individual points.

At median 2.3-year follow-up (1.0-4.8), all FFI and FAAM subscores improved significantly (*P* < .001 for all). MCID achievement, detailed in Figure 3, was FFI 36 of 43 (84%), FFI-Global 40 of 43 (93%), FAAM ADL Index 34 of 42 (81%), FAAM ADL Global 36 of 42 (86%), FAAM Sport Index 34 of 42 (81%), and FAAM Sport Global 33 of 43 (79%). Patients reported being "satisfied" or "very satisfied" with 33 of 43 ankles (77%) (Figs 3 and 4). Compared with arthroscopic confirmation of syndesmotic widening ≥2 mm, imaging sensitivity was 5% for weight-bearing radiographs (1/21), 22% for bilateral CT (5/23), and 26% for MRI (10/38) (Table 1).

**Fig 5:**
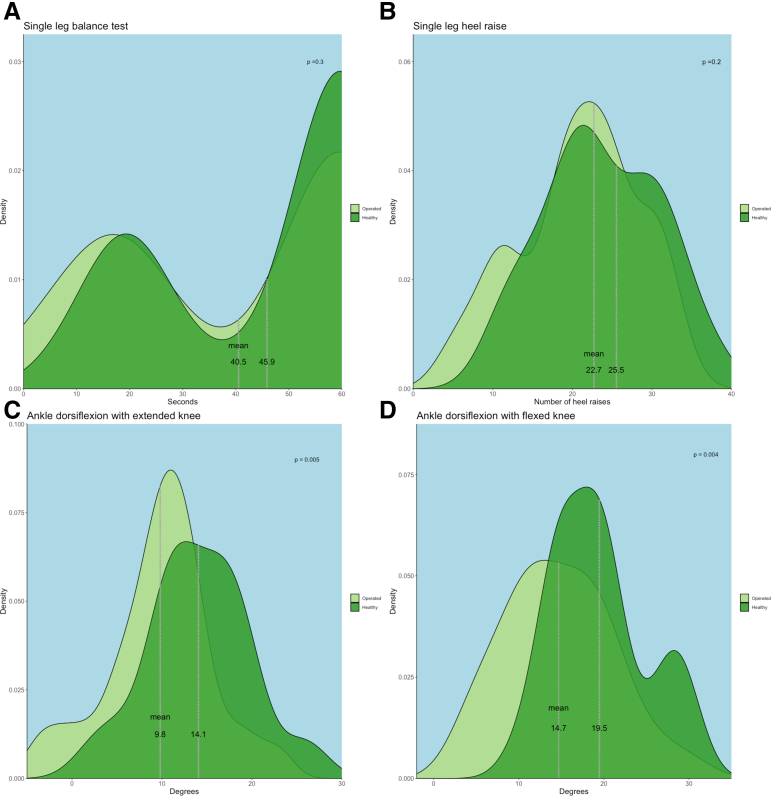
Functional test results: Comparison of functional outcomes between operated ankles (light green) and healthy, nonoperated ankles (dark green). No significant differences were observed in single leg balance (A: 40.5 seconds vs 45.9 seconds, *P* = .3) or heel raise endurance (B: 22.7 vs 25.5 repetitions, *P* = .2). However, ankle dorsiflexion was significantly reduced in operated ankles with both extended (C: 9.8° vs 14.1°, *P* = .005) and flexed knees (D: 14.7° vs 19.5°, *P* = .004).

Functional testing in 31 patients (33 operated, 29 contralateral ankles) showed no significant differences in single-leg balance (*P* = .30) or heel raise endurance (*P* = .20). Contralateral ankles had 4 to 5° greater dorsiflexion (*P* = .005 knee-extended, *P* = .004 knee-flexed, Fig 5). Radiographic comparison revealed comparable medial clear space (operated 2.4 mm vs nonoperated 2.5 mm; mean difference 0.1 mm, 95% confidence interval −0.1 to 0.2, *P* = .21) and talus-tibia plafond distance (both 3.5 mm; mean difference 0.1 mm, 95% confidence interval 0.0-0.2, *P* = .11). Anterior tibial bony spurs were present in 10 of 33 operated ankles (30%) and absent in all nonoperated ankles.

Complications occurred in 4 of 43 ankles (9%): one deep infection requiring implant removal, two recurrent instabilities treated with new suture-buttons (one post-trauma, one after elective removal), and one sural-nerve injury managed conservatively with Botox injections.

Elective implant removal for stiffness or irritation was performed in 15 of 43 ankles (35%). Five additional procedures were undertaken: 3 at index surgery (peroneus-brevis tubulation, tarsal-tunnel decompression, single talus microfracture) and 2 later (tibial-cyst grafting, deltoid-ligament re-attachment).

## Discussion

Arthroscopic suture-button repair of the syndesmosis produced significant improvements in every FFI and FAAM subscore at a median 2.3-year follow-up, with 79-93% of ankles reaching a stringent MCID. Two additional points merit emphasis. First, 15 of 39 ankles (38%) showed a posterior-predominant (“reverse open-book”) widening pattern on arthroscopy, a finding infrequently reported in earlier series.^13^ Second, standard imaging modalities—weight-bearing radiographs, stress fluoroscopy, CT, and MRI—had poor sensitivity (≤ 26%) for detecting these chronic injuries. Collectively, these observations highlight the under-recognized clinical importance of subtle, especially posterior, syndesmotic instability and indicate that current diagnostic algorithms often fail to identify these injuries.

### Re-Examining the Concept of Ankle Instability

Traditionally, mechanical instability hinges on talar varus tilt, while persistent symptoms without tilt are labeled "functional" instability.^24^^,^^25^ In our cohort, however, most ankles with arthroscopically confirmed syndesmotic widening had ≤5° of tilt on stress fluoroscopy, and only 13 of 34 ankles (38%) had a positive Cotton test. Absent arthroscopy these injuries would likely have been considered "functional." By prioritizing a clear syndesmotic-injury history plus >3 months of disabling pain and dysfunction over isolated signs or imaging, our protocol uncovered this otherwise occult pathology.

Although some question its usefulness, stress radiography remains integral to our algorithm.^26^ A talar tilt ≥10 to 15° on stress films indicates lateral ligament instability and warrants stabilization, whereas minimal tilt (≤5°) should instead prompt syndesmotic evaluation rather than functional attribution.^27^ Early red flags—prolonged non–weight-bearing, extensive medial/proximal bruising, and marked early swelling—heighten suspicion for syndesmotic injury.^4^, ^5^, ^6^ In chronic presentations, persistent swelling, stiffness, push-off weakness, and subjective instability, with normal stress tests, are common.^6^^,^^10^^,^^11^ Clinical patterns further refine the diagnostic workup where intermittent, pain-free intervals between sprains suggest lateral instability, whereas ongoing pain despite rest points toward syndesmotic pathology.^28^ Accordingly, we perform diagnostic arthroscopy of the syndesmosis even when stress radiographs favor lateral repair, unless the clinical history and examination clearly exclude syndesmotic involvement.

### Reinterpreting Syndesmotic Findings in the Literature

Previous studies that centered on lateral instability have frequently overlooked chronic syndesmotic injury. Su et al. diagnosed lateral instability using history, recurrent giving-way, and positive anterior-drawer or manual-varus tests—supplemented by MRI evidence of anterior talofibular and calcaneofibular ligament injuries—but did not employ talar-tilt radiography.^19^^,^^29^^,^^30^ Although their systematic arthroscopies revealed syndesmotic diastasis ≥4 mm in several ankles, all patients underwent lateral-only stabilization, an approach linked to poorer outcomes in that subgroup.^29^

Vega et al.^31^ likewise found posterior syndesmotic injury in over half of ankles clinically labeled as “functional" instability. However, performing the posterior part of the arthroscopy under traction likely compressed the syndesmosis, masking widening and redirecting focus to subtle lateral-ligament irregularities, thus spawning the “lateral micro-instability” concept.^32^

By contrast, our traction-free arthroscopic technique consistently revealed a posterior-predominant (“reverse open-book”) widening pattern alongside anterior syndesmotic tenderness. In our chronic cohort (median 1.7 years of failed nonoperative care), suture-button stabilization of even modest syndesmotic widening (≥2 mm) produced significant improvements in validated PROMs, thereby challenging cadaveric and acute-injury reservations about the 2 mm threshold and extending beyond earlier AOFAS-based criteria of ≥4 mm.^16^^,^^29^^,^^33^, ^34^, ^35^, ^36^

Together, our findings and these reinterpretations underscore that chronic syndesmotic instability, not isolated lateral-ligament disease, often drives ankle dysfunction mischaracterized as “functional” instability, and they make a compelling case for routine arthroscopic evaluation and syndesmotic stabilization.

### Poor Diagnostic Performance of Standard Imaging and Underdiagnosis

In our cohort of chronic syndesmotic injuries, standard imaging performed poorly. MRI regarded as the soft-tissue gold standard failed to detect syndesmotic pathology in 74% of ankles (28/38), despite its well-documented sensitivity in acute settings.^14^^,^^15^ This discrepancy reflects likely chronic remodeling: ligaments remain structurally intact on imaging yet exhibit functional laxity, revealed only by arthroscopic tibiofibular separation. Likewise, Su et al. reported anterior talofibular and calcaneofibular ligament injuries detected on MRI but made no mention of syndesmotic injuries, even though their arthroscopies confirmed widening in multiple ankles.^29^ Other modalities fared no better—stress fluoroscopy, weight-bearing radiographs, and axial CT showed sensitivities of merely 5% to 22%. Together, these findings highlight the underdiagnosis of chronic syndesmotic injuries when relying on standard imaging and support a lower threshold for diagnostic arthroscopy.^6^^,^^14^

Underperformance of conventional imaging, the misclassification of painful ankles without measurable talar tilt as “functional” problems, and the often-posterior predominance of chronic diastasis likely explain why syndesmotic instability remains underdiagnosed. Although syndesmotic injuries may account for up to 25% of ankle sprains, fewer than 200 operative chronic cases have been reported.^13^ In our 4-year series (43 ankles), the captured operative incidence translates to approximately 2 to 3 per 100,000 person-years, suggesting that the true burden of chronic syndesmotic pathology is substantially greater.

### Surgical Management, Timing, and Clinical Implications

Despite a 35% implant-removal rate for irritation or stiffness and a 30% prevalence of anterior tibial spurs on follow-up radiographs, ankles treated with our technique matched their contralateral controls in balance and endurance tests and maintained radiographic congruency. Day-case surgery with 2 suture-buttons, immediate mobilization, and progression to full weight-bearing from 3 weeks produced substantial PROM gains with a low major-complication rate. Importantly, these favorable results were achieved with suture-buttons used as the primary stabilization, without biological graft augmentation, underscoring the value of this technique as a standalone intervention for this challenging condition. Hence, when conservative care has failed, this surgical strategy appears both efficient and safe.

However, the timing of surgery for chronic syndesmotic instability requires careful balance. In our cohort, the median interval from injury to repair was 1.7 years, and most patients had been symptomatic for >6 months. Although we set ≥3 months of symptoms as an inclusion threshold to confirm chronicity, our broader experience now leans towards an initial 4- to 6-month period of rehabilitation. Meaningful improvement justifies continued nonoperative care. If symptoms plateau or worsen despite adequate therapy, earlier surgery is warranted to avert protracted disability. Notably, symptom duration in our series ranged from 3 months to 25 years, yet positive outcomes were observed across this spectrum. This suggests that arthroscopic suture-button repair can remain effective even in exceptionally long-standing injuries, offering hope to patients who have endured symptoms for many years.

The pronounced PROM gains (79%-93% achieving MCID) and the 77% satisfaction rate reinforce the clinical value of identifying and treating this previously largely occult condition. Yet, recent comprehensive reviews of lateral ankle instability overlook chronic syndesmotic injury, illustrating a persistent knowledge gap.^27^

Maintaining a high index of suspicion—and proceeding to arthroscopy when the history and persistent symptoms implicate the syndesmosis, can spare patients from being consigned indefinitely to the “functional’’ category or undergoing lateral-ligament surgery for a misdiagnosis that risks lifelong dysfunction.

### Limitations

Our study has several important limitations. First, the retrospective, single-center design and absence of a nonoperative control group limit both causal inference and generalizability.

Second, our median follow-up of 2.3 years provides valuable midterm data, but long-term outcomes remain unknown.

Imaging sensitivity estimates may be skewed by nonstandardized protocols across modalities, small sample sizes (especially for CT and bilateral weight-bearing radiographs), and reliance on final radiology reports rather than re-evaluation of source images. Moreover, variable emphasis on syndesmotic injury, particularly in MRI requisitions, reflects real-world practice but may have further affected diagnostic accuracy.

Treatment-related confounders also warrant consideration. Fifteen of 43 ankles (35 %) underwent concomitant modified Broström repair, making it difficult to isolate the effect of syndesmotic stabilization alone. Elective suture-button removal for stiffness or irritation (15/43, 35 %) and a 30% incidence of anterior tibial spurs temper our otherwise favorable PROM gains and may relate, in part, to our decision to stabilize syndesmotic widening as small as ≥2 mm. While we applied a stringent MCID threshold (75% of the preoperative standard deviation), validated MCID values specific to chronic syndesmotic instability have yet to be established.

A single permanent sural-nerve injury—likely sustained during posterior syndesmosis debridement in an ankle with minimal talar tilt—highlights potential morbidity. We have since modified our technique to direct the shaver medially in such cases and to perform debridement less aggressively. Finally, we did not review arthroscopies performed for other indications, so asymptomatic widening of 2 to 4 mm in ankles without syndesmotic complaints cannot be excluded. If such cases exist, they could lead us to overestimate the clinical relevance of the 2 mm threshold.

## Conclusions

Arthroscopic suture-button repair led to significant improvements in patient-reported outcomes for chronic isolated syndesmotic injuries. Standard imaging had low sensitivity, and a posterior-predominant syndesmotic widening pattern was commonly observed arthroscopically. These findings suggest that chronic syndesmotic instability is underdiagnosed, highlight the role of arthroscopic evaluation for accurate diagnosis, and support the use of suture-button stabilization as an effective treatment.

## Declaration of Generative AI and AI-assisted technologies in the writing process

During the preparation of this work the author(s) used multiple AI platforms—ChatGPT (OpenAI), Claude (Anthropic), DeepSeek, Gemini (Google), Grok (x.ai), and Perplexity AI—for language refinement, systematic literature synthesis, and coding support (R/Python). These tools also facilitated discussion of ankle instability concepts and helped refine the Discussion. All study design, data analysis, interpretation, and scientific conclusions remained the authors’ responsibility. The authors critically reviewed and edited every AI-assisted element and accept full accountability for the integrity of this work.

## Disclosures

The authors declare the following financial interests/personal relationships which may be considered as potential competing interests: R.M.H. reports paid lectures for the following companies: Arthrex, Smith & Nephew, and Depuy Synthes, all shoulder-related. All other authors (T.V., E.I, J.J., N.V., M.A.M., K.M.) declare that they have no known competing financial interests or personal relationships that could have appeared to influence the work reported in this paper.
